# Mycobacterial DNA Extraction for Whole-Genome Sequencing from Early Positive Liquid (MGIT) Cultures

**DOI:** 10.1128/JCM.03073-14

**Published:** 2015-03-18

**Authors:** Antonina A. Votintseva, Louise J. Pankhurst, Luke W. Anson, Marcus R. Morgan, Deborah Gascoyne-Binzi, Timothy M. Walker, T. Phuong Quan, David H. Wyllie, Carlos Del Ojo Elias, Mark Wilcox, A. Sarah Walker, Tim E. A. Peto, Derrick W. Crook

**Affiliations:** a Nuffield Department of Clinical Medicine, University of Oxford, John Radcliffe Hospital, Oxford, United Kingdom; b Department of Microbiology, Leeds General Infirmary, Leeds, United Kingdom; c National Institute for Health Research Oxford Biomedical Research Centre, John Radcliffe Hospital, Oxford, United Kingdom; d The Jenner Institute, University of Oxford, Oxford, United Kingdom; e Microbiology Laboratory, John Radcliffe Hospital, Oxford University Hospitals NHS Trust, Oxford, United Kingdom

## Abstract

We developed a low-cost and reliable method of DNA extraction from as little as 1 ml of early positive mycobacterial growth indicator tube (MGIT) cultures that is suitable for whole-genome sequencing to identify mycobacterial species and predict antibiotic resistance in clinical samples. The DNA extraction method is based on ethanol precipitation supplemented by pretreatment steps with a MolYsis kit or saline wash for the removal of human DNA and a final DNA cleanup step with solid-phase reversible immobilization beads. The protocol yielded ≥0.2 ng/μl of DNA for 90% (MolYsis kit) and 83% (saline wash) of positive MGIT cultures. A total of 144 (94%) of the 154 samples sequenced on the MiSeq platform (Illumina) achieved the target of 1 million reads, with <5% of reads derived from human or nasopharyngeal flora for 88% and 91% of samples, respectively. A total of 59 (98%) of 60 samples that were identified by the national mycobacterial reference laboratory (NMRL) as Mycobacterium tuberculosis were successfully mapped to the H37Rv reference, with >90% coverage achieved. The DNA extraction protocol, therefore, will facilitate fast and accurate identification of mycobacterial species and resistance using a range of bioinformatics tools.

## INTRODUCTION

Technological advances over the past 20 years have led to the widespread use of molecular assays that aid the diagnosis of tuberculosis (1–6). These assays are able to rapidly identify the organism Mycobacterium tuberculosis to the species level and can also identify a small number of common drug resistance-conferring mutations. The sensitivity of these molecular assays for detecting drug resistance has been limited by design, and phenotyping remains the gold standard. The low growth rate of M. tuberculosis ensures that the confirmatory phenotype still takes weeks or months to obtain. The number of routine tests currently performed to identify mycobacterial species, determine drug susceptibilities, and generate a molecular profile for purposes of surveillance means that the diagnostic process remains not just slow but also expensive (7–9).

Whole-genome sequencing (WGS) is rapidly being established as a high-resolution method of linking cases to outbreaks by identifying single nucleotide polymorphisms (SNPs) with advantages over current fingerprinting methods (10–14). The excellent reproducibility means WGS also has the potential as a diagnostic test to identify species and as many drug resistance-conferring mutations as might be defined. As the costs of WGS are now comparable to the costs of molecular fingerprinting, the prospect of deriving additional results on species identity and drug resistance from the same sequence data at no additional cost is financially appealing. Were WGS to produce results faster than current culture-based methods, such an approach would also be attractive from a clinical point of view. WGS is already used routinely in a number of clinical and public health laboratories locally (15–19) and worldwide (http://www.globalmicrobialidentifier.org/).

It would be optimal to produce a high-quality whole-genome sequence from primary clinical specimens, but sequencing M. tuberculosis directly from sputum samples is currently able to achieve only 0.002× to 0.7× coverage of the reference genome due to high contamination with human DNA (up to 99% of reads) (20). Therefore, current technology still requires an initial culture step to ensure reproducibility. The Bactec mycobacterial growth indicator tube (MGIT) (Becton Dickinson, United Kingdom) automated liquid culture system is widely used to culture most clinically relevant mycobacterial species. Although it is standard practice to pretreat clinical samples to reduce overgrowth by other bacteria and fungi prior to MGIT inoculation, human and bacterial DNA are still likely to contaminate the culture. Here, we describe a method developed for extracting and purifying mycobacterial DNA for whole-genome sequencing from MGIT tubes within hours to days of culture positivity.

## MATERIALS AND METHODS

### Sample selection and processing.

Consecutive positive MGIT cultures were taken from isolates of patients referred to the microbiology departments at the John Radcliffe Hospital, Oxford (*n* = 204), and the Leeds General Infirmary (*n* = 31) as part of routine clinical care. Prior to culturing, all respiratory samples and other samples from nonsterile sites were decontaminated with a final concentration of either 2% sodium hydroxide (Oxford) or 3% sodium hydroxide (Leeds) for 30 min or 15 min, respectively. Respiratory samples from patients with cystic fibrosis were treated with 5% oxalic acid for 30 min (Oxford) or 90 min (Leeds). Specimens from normally sterile sites were not decontaminated unless they were known to be positive for other bacteria. A 1-ml aliquot of liquid culture was taken from the base of the tube as soon after culture positivity as feasible but only after sufficient culture material had been obtained for the routine diagnostic workflow. Special care was taken to sample mycobacterial growth (seen as crumbs) within the 1-ml aliquot. Aliquots were transferred to 2-ml screw-cap tubes and heat inactivated in a thermal block after sonication (for 15 min at 35 kHz) initially for 2 h at 95°C but reduced to 30 min at 95°C after this shorter time period had been validated as effective at mycobacterial inactivation. (Thirty-six M. tuberculosis cultures were heat killed 1 to 7 days after positivity and reincubated, with all remaining cultures negative at 49 days.)

### Mycobacterial DNA extraction.

Two column-based DNA extraction methods (QIAamp DNA minikit [Qiagen, Germany] and QuickGene DNA tissue kit S for QuickGene-Mini80 [QG] [Kurabo, Japan]) were tested, each with and without a pretreatment step using the MolYsis Basic5 kit (Molzym, Germany) to remove human DNA. The samples that were pretreated with the MolYsis kit were processed following the manufacturer's protocol. After the pretreatment step, the manufacturer's protocol for the QIAamp DNA minikit method of DNA purification from blood or body fluids (Spin Protocol) was followed, and a modified DNA tissue kit S manufacturer's protocol for the QG method was followed.

**FIG 1:**
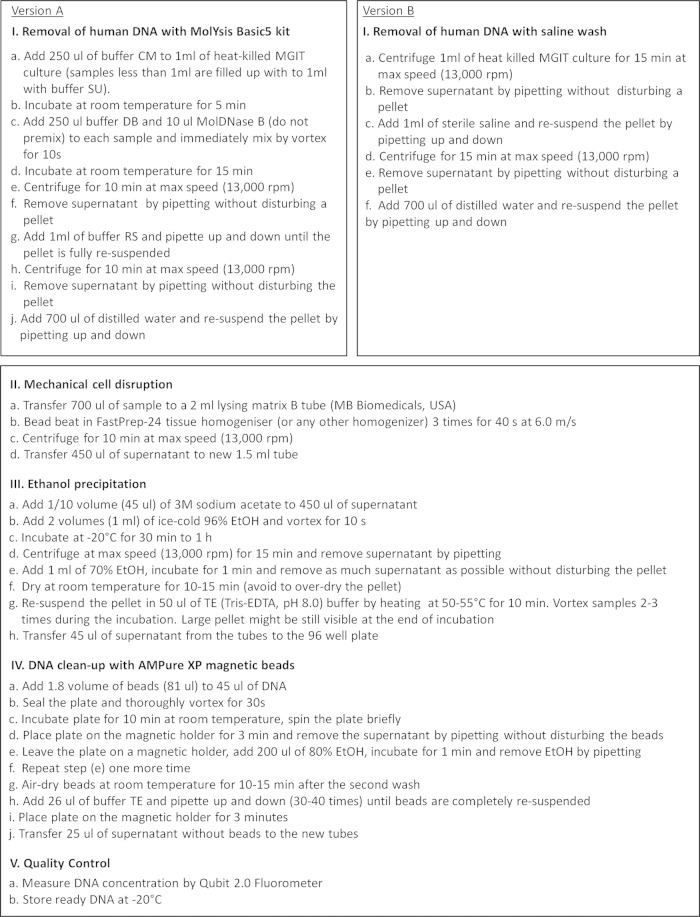
Protocol for mycobacterial DNA extraction from the MGIT cultures by ethanol precipitation with the MolYsis Basic5 kit (version A) or saline wash (version B) pretreatment steps for removal of human DNA.

A third, non-kit-based, extraction method based on ethanol precipitation was also tested using either the MolYsis kit or a saline wash as the pretreatment. Figure 1 shows the final version of the ethanol precipitation protocol for the DNA extraction from early positive MGIT cultures.

Following all extractions, DNA was purified using AMPure XP solid-phase reversible immobilization (SPRI) beads (Beckman Coulter, United Kingdom) to remove inhibitors with the potential to interfere with sequencing library preparation (see below). DNA was eluted finally in 26 μl 1× Tris-EDTA (TE) (pH 8.0) buffer.

After all three extraction methods were completed, the DNA concentrations were measured using the Qubit 2.0 fluorometer (LifeTechnologies, USA). Extractions yielding concentrations of ≥0.2 ng/μl DNA (the required input for Nextera XT [Illumina, USA] library preparation) were considered successful.

### Real-time PCR.

Quantification of human DNA in samples extracted with and without the MolYsis version of the QIAamp protocol was performed using human β-actin primers, a β-actin probe, and the reaction condition described in Herrera at al., in 2005 (21). Real-time PCR was performed in triplicate for each sample and for a standard and a negative template control using Stratagene Mx3005P (Agilent Technologies, USA).

### Library preparation and Illumina MiSeq sequencing.

Libraries were prepared for the MiSeq sequencing using the Illumina Nextera XT protocol (Illumina, part 15031942 rev. C, October 2012). The original Nextera XT protocol resulted in weak libraries (<5 ng/μl) and uneven sample coverage during the MiSeq runs. To increase the DNA library concentrations, the index PCR amplification program on the thermal cycler (manufacturer's instructions, step 10, p 27) was extended from 12 to 15 cycles. To ensure evenness of sample coverage, the magnetic bead-based library normalization step (manufacturer's instructions, p 34 to 38) was replaced by a manual library normalization step, based on the library's DNA concentration and average size, as measured by the Qubit and 2200 TapeStation (Agilent Technologies, USA). For the detailed version of the modified Nextera XT library protocol, see Protocol S1 in the supplemental material.

The MGIT samples prepared during the development of the DNA extraction method, and while adjustments of the Nextera XT protocol were undertaken, were sequenced as MiSeq test runs, while samples prepared by the final DNA extraction and final Nextera XT protocols were sequenced as MiSeq live runs. The samples were batched 12 to 16 per flow-cell, and paired-end sequencing was performed using the MiSeq reagent kit v2, with 2 × 150 bp and with the H37Rv M. tuberculosis reference genome included as a technical replicate on each live run. The 12 to 16 samples as the batch size was determined by the number of culture-positive samples available from the routine laboratory. Batching enabled us to run the MiSeq once every 2 weeks. When the study was started, the MiSeq reagent kit v3 was not available; we kept the v2 kit throughout to avoid introducing potential bias. We chose the 300-bp over the 500-bp v2 kit, because it was faster (24 h versus 39 h, respectively), and our prior experience in sequencing cultured mycobacterial isolates demonstrates that 150-bp-long reads are sufficient for bioinformatic analysis of mycobacterial species (12, 13).

### Bioinformatics analysis.

Illumina reads were mapped to the M. tuberculosis H37Rv reference strain using Stampy v1.22, and the variants were called using Samtools v0.1.18. The reference genome was masked, removing repeated regions by using a self-self blast approach. Only the variants with ≥5 high-quality reads, a mean quality per base ≥25, and >90% high-quality bases were retained as variants; the positions called heterozygous by Samtools based on >10% of a second variant present were not retained.

Each DNA extraction was first assessed in terms of the number of reads, with at least 1 million reads considered the minimum necessary for downstream bioinformatics processing. This arbitrary threshold was chosen on the basis that, when contamination level was low (≤5% to 10% of reads), 1 million was the minimum number of reads for which the M. tuberculosis complex (MTBC) study samples obtained ≥90% coverage of the reference genome.

Sequences were then assessed for contamination with human DNA and DNA from nasopharyngeal flora (NPF). Human DNA contamination was estimated by mapping the reads to the human genome GRCh37 (hg19), and identified reads were discarded. The NPF DNA was identified by aligning the reads to nasal, oral, and mouth flora available in the NIH human microbiome project database (http://www.hmpdacc.org/). The MTBC samples with ≥1 million reads successfully mapped to ≥90% of the reference genome even with 5% to 10% of contaminating reads. Therefore, a contamination rate of <5% for each of the human and NPF DNA was considered acceptable.

### Statistical analysis.

Multivariate linear regression was used to identify independent factors affecting DNA concentration. Model selection was performed by backwards elimination on all factors with an exit *P* value of 0.05. Interactions between the final model variables were checked and included if the *P* value was <0.05. Analyses were performed using Stata 13.1 (Stata Corp., USA).

We sought and received advice from local research governance that no institutional ethical review was required, because this was a laboratory study using only bacterial DNA extracted from samples identified only by laboratory numbers with no personal or clinical data.

### Nucleotide sequence accession numbers.

Genome sequence data have been deposited to the Sequence Read Archive (SRA), NCBI, under the BioProject ID 268101 and the samples accession numbers SAMN03225240 through SAMN03225393.

## RESULTS AND DISCUSSION

A total of 204 positive MGIT cultures were obtained from Oxford, and 31 were obtained from Leeds. All cultures were heat inactivated between 0 to 17 days (median, 3 days; interquartile range [IQR], 2 to 5 days) after culture positivity.

### DNA extraction.

**FIG 2:**
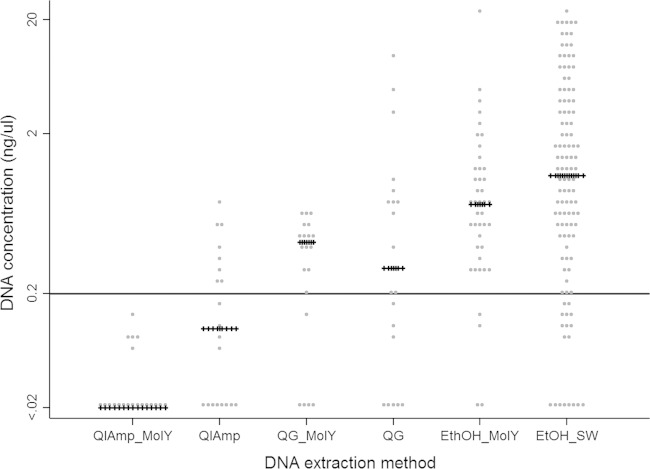
DNA extraction from early positive MGIT cultures with QIAmp minikit with MolYsis (QIAmp-MolY) and QIAmp minikit (QIAmp), QuickGene with MolYsis (QG-MolY) and QuickGene (QG), ethanol precipitation protocol with MolYsis (EtOH-MolY), and ethanol precipitation protocol with saline wash (EtOH-SW). Each gray dot represents a single extraction; crossed horizontal lines represent median DNA concentration for each extraction method; solid black line represents the threshold amount of DNA of 0.2 ng/μl required for the MiSeq library preparation.

A total of 40 MGIT cultures were extracted using the QIAamp kit, and 40 were extracted using the QG kit. In each case, 20 cultures were pretreated by MolYsis and 20 were not. The QIAamp method achieved 8/20 (40%) successful extractions without MolYsis pretreatment compared to 0/20 (0%) with MolYsis pretreatment (*P* = 0.03) (Fig. 2). The QG method achieved 13/20 (65%) and 10/20 (50%) successful extractions with and without MolYsis, respectively (*P* = 0.52). The relative success of pretreatment in removing the human DNA was assessed using real-time PCR in samples extracted with (*n* = 3) and without (*n* = 3) the MolYsis version of the QIAamp protocol. The samples extracted without pretreatment contained a variable amount of human DNA (999, 112, and 9 copies/μl), while the addition of a MolYsis step successfully reduced the human DNA to below the detection limit of the assay (i.e., 0 copies/μl detected) in all extracts.

As DNA extraction using kit-based methods was not considered effective, an alternative ethanol precipitation method was successfully tested and used to process all additional samples. As real-time PCR confirmed the widespread contamination with human DNA after kit-based extractions without MolYsis pretreatment, MolYsis was initially applied to subsequent extractions. However, a saline wash was tested as a possible alternative pretreatment step to the expensive and time-consuming MolYsis kit. A total of 154 samples (median culture age, 4 days; IQR, 2 to 6 days) were processed, of which 42 (27%) were pretreated with MolYsis and 112 (73%), with a saline wash. Overall, the DNA yield was greater than that for the kit-based methods, with 38/42 (90%) successfully meeting the ≥0.2 ng/μl DNA concentration threshold after MolYsis pretreatment and 93/112 (83%) meeting the threshold after pretreatment with a saline wash (Fig. 2). There was no evidence that the DNA yield depended on the specific pretreatment (*P* = 0.32). As the saline wash is cheaper and less time consuming than the MolYsis kit, a saline wash was chosen over MolYsis for inclusion in the final protocol.

The success rate of 83% of extractions with the saline wash version of the ethanol precipitation protocol was achieved on a limited amount (1 ml) of MGIT culture. It would be expected that the success rate should improve if larger volumes could be used. Indeed, on the one occasion we were able to extract DNA using the entire MGIT volume (7 ml), it yielded a total of 733 ng DNA at 48 h after culture positivity.

To investigate factors affecting DNA extraction, a multivariate linear regression model for absolute DNA yield (ng/μl) was constructed. Six factors were considered independent predictors: (i) sample type (sputum, bronchoalveolar lavage fluid, tissue, pus, or aspirate), (ii) age of MGIT culture prior to heat inactivation (days from positivity), (iii) heat inactivation time (30 min or 2 h), (iv) days between heat inactivation and extraction, (v) mycobacterial species (M. tuberculosis complex, Mycobacterium avium complex, Mycobacterium abscessus complex, or others), and (vi) growth rate of mycobacteria (high or low, as described by Tortoli et al. [22]). Only the age of the culture prior to heat inactivation, heat inactivation time, and growth rate of mycobacteria independently significantly affected the DNA yield, with no significant interactions between any of these factors (Table 1). As expected, the 2-h heat inactivation step was associated with a lower DNA yield from extraction than the 30-min protocol (*P* = 0.01), an effect likely related to limiting the degradation of DNA during heat inactivation. A longer time from MGIT culture positivity to heat inactivation also increased the DNA yield (*P* = 0.01), as did fast-growing mycobacteria compared to slow growers (*P* = 0.03). Again, these results were expected, as the greater biomass cultured with time would be expected to yield more DNA. Even if early heat inactivation from the moment of MGIT positivity resulted in a lower DNA yield, it would not compromise the quality of WGS, as only 0.2 ng/μl is required for the library preparation. When possible, the visible mycobacteria growths (i.e., the crumbs) were collected within the 1-ml aliquot from each MGIT, which is likely to have contributed to these successful early extractions.

**TABLE 1:** Factors that affect DNA yield from the early positive MGIT cultures

Factor	Impact on DNA yield (ng/μl)	*P*	95% CI^*a*^
Age of MGIT culture, per additional day	0.131	0.01	−0.233 to 0.029
Heat inactivation time, 2 h versus 30 min	−0.838	0.01	−1.486 to −0.191
Speed of growth, slow versus fast	0.994	0.03	−1.901 to 0.087

a CI, confidence interval.

### MiSeq sequencing.

In total, 170 samples were extracted with the ethanol precipitation protocol, including 154 initial extractions and 16 reextracted samples. A total of 23 (15%) of the 154 initial extractions yielded <0.2 ng/ml DNA. Nine of the 23 samples were not reextracted, because no MGIT cultures were available (3 samples) or because they had <0.2 ng/μl DNA and were successfully sequenced during the testing for a low DNA limit for MiSeq library preparation (6 samples). The other 14 initial extraction failures were reextracted after an additional 5 days of growth, as were 2 pus samples that initially yielded ≥0.2 ng/μl DNA but required reextraction after MiSeq sequencing, as they were heavily contaminated with human DNA (42% to 85%). For most samples (93%, 13/14) that initially yielded <0.2 ng/μl DNA, reextraction after additional culturing significantly improved DNA yield to 0.3 to 57 ng/μl. Only one sample failed reextraction, with no DNA detected. However, 4/13 samples reextracted because of a low initial DNA yield had to be reextracted again after MiSeq sequencing, as they had high levels of contamination or low read numbers.

Overall, 9% (16/170) of the DNA extractions were not sequenced on MiSeq because of low DNA amounts after extraction (13 samples) or failed reextraction (1 sample), or because they were used to adjust the library preparation protocol (2 samples).

In total, 154 samples were sequenced on the MiSeq platform on test (*n* = 60) and live (*n* = 94) runs, including 6 samples that were sequenced twice. Four of 6 samples were resequenced, as they had insufficient read numbers, and 2/6 pus samples were resequenced, as they were heavily contaminated with human DNA (42% to 85%). Seven of 60 test and 3/94 live samples had <0.2 ng/ml DNA (the target concentration for Nextera XT library preparation) after extraction.

The target of >1 million reads was achieved for 144/154 (94%) isolates, with 80% (8/10) of the samples below the 1 million read thresholds being sequenced on test runs during the library preparation development phase. Most (6/8) of these test run samples with low read numbers had >0.2 ng/ml DNA sequenced, suggesting that low read numbers were due to the library protocol used in test runs. Low-level (<5%) contamination with human or NPF DNA was observed for 136/154 (88%) and 140/154 (91%) samples, respectively (Table 2), suggesting successful decontamination by both pretreatment protocols. Three of 4 samples containing >40% human DNA were cultured from the pus and tissue samples. The successful fractionation of the NPF and mycobacterial DNA may also have been aided by pretreating the primary sample with 2% to 3% sodium hydroxide in final solution before MGIT inoculation, while treatment with a lower concentration of sodium hydroxide has been shown to result in higher proportions of NPF reads (23). While the thresholds we used are arbitrary, it would be reasonable to expect that lower read numbers and higher proportions of contaminating reads would impair the accuracy of downstream bioinformatics analysis.

**TABLE 2:** Total read numbers and proportions of human and nasopharyngeal flora reads in test and live run samples

Category	Samples (no. [%]) for indicated group^*a*^		Total per category
	Test runs	Live runs	
Number of reads			
<1 million	8 (13)	2 (2)	10 (6)
1–2 million^*b*^	17 (28)	12 (13)	29 (19)
2–3 million	16 (27)	45 (48)	61 (40)
3–4 million	12 (20)	22 (23)	34 (22)
>4 million	7 (12)	13 (14)	20 (13)
No data	0	0	0
Proportion of human reads			
0–5%^*c*^	50 (83)	86 (92)	136 (88)
6–20%	2 (3)	4 (4)	6 (4)
21–30%	1 (2)	1 (1)	2 (1)
31–40%	0	0	0
>41%	1 (2)	3 (3)	4 (3)
No data	6 (10)	0	6 (4)
Proportion of nasopharyngeal reads			
0–5%^*c*^	48 (79)	92 (98)	140 (91)
6–20%	1 (2)	2 (2)	3 (2)
21–30%	2 (3)	0	2 (1)
31–40%	2 (3)	0	1 (1)
>41%	2 (3)	0	2 (1)
No data	6 (10)	0	6 (4)

a The total number of test and live run samples was 154.

b Indicates a target coverage of 1 million reads required for data analysis.

c Indicates a target threshold for contamination with human/nasopharyngeal flora DNA. Fisher's exact test (test versus live runs) for sample coverage (*P* = 0.003), proportion of human DNA (*P* = 0.02), and proportion of nasopharyngeal flora DNA (*P* < 0.001). All *P* values support superior performance of the final protocol in the live versus test runs.

To further assess the utility of isolating and purifying mycobacterial DNA from early positive MGITs, the 60 sequenced samples which had been identified by the national mycobacterial reference laboratory (NMRL) as MTBC using standard methods were mapped to the H37Rv reference genome. Overall, 39/40 (98%) samples directly identified as MTBC by NMRL mapped successfully to H37Rv, with ≥90% of the reference genome covered (median, 91.8%; IQR, 91.5% to 92%; range, 89.2% to 92.1%), as did all 20 samples cross-referenced to another clinical isolate identified as MTBC from the same patient episode (median, 91.8%; IQR, 90.8% to 91.9%; range, 91.9% to 92.1%). One sample had a reference genome coverage of only 2.8%, based on 2.6 million reads, with contamination of 89% of human reads and <1% of NPF reads. The sample was cultured from pus, accounting for the high percentage of human DNA contamination. The sample was reported by the NMRL as a mixture of MTBC and Mycobacterium intracellulare.

To test the lower limits of DNA concentration from which Nextera XT can successfully produce libraries, 8 samples with DNA concentrations between 0.06 and 0.17 ng/μl were prepared. All 8 sequenced isolates produced >2 million reads, of which <10% were human reads. However, 2/8 (25%) samples, with DNA concentrations of 0.06 to 0.12 ng/μl, were largely contaminated by NPF (>50% of reads). These two samples, identified as MTBC by NMRL, had reference genome coverage of only 1.6% and 1.1%, respectively. Both samples were reextracted after additional incubation and resequenced. Resequencing showed that additional incubation reduced the amount of NPF flora contamination to <5% and improved the reference genome coverage to >90%. However, another two samples with concentration of 0.08 and 0.1 ng/μl DNA that were identified by NMRL as MTBC had reference genome coverage of 92% and 90%, respectively. This demonstrates that samples with as little DNA as 0.08 to 0.1 ng/μl can be successfully sequenced on the MiSeq. Further, as described above, two samples that yielded no detectable DNA were processed and sequenced on MiSeq test runs. These samples actually produced reads of 0.26 and 0.27 million, respectively, of which <5% were NPF reads. One sample had 19%, and another had <5%, human reads. Surprisingly, the two samples achieved reference genome coverage of 56% and 68%, respectively. These two samples were identified by NMRL as MTBC and were reextracted after additional culturing and resequenced, which improved the reference genome coverage to >90%. This demonstrates the potential for less than 0.5 ng/ml of DNA, corresponding to the low detectable limit for the Qubit dsDNA HS assay kit (LifeTechnologies, USA), to be sequenced on MiSeq.

In summary, our modified Nextera XT protocol enables WGS from MGIT extracts with DNA concentrations as low as 0.08 ng/μl and strong libraries to be consistently achieved from concentrations of 0.2 ng/μl or above. Decontamination of clinical samples with 2% to 3% sodium hydroxide prior to MGIT inoculation is important to reduce NPF flora contamination. Pretreatment of positive culture with MolYsis or a saline wash prior to extraction is essential to reduce human DNA contamination. WGS data could be obtained within 3 days from the moment a MGIT culture flagged positive and will facilitate fast and accurate diagnosis of mycobacteria in the future.

## Supplementary Material

Supplemental material
