# Supplementary material for: Mycobacterial DNA Extraction for Whole-Genome Sequencing from Early Positive Liquid (MGIT) Cultures
Source: J Clin Microbiol. 2015 Mar 18;53(4):1137–43. doi: 10.1128/JCM.03073-14 (PMC4365189; doi:10.1128/JCM.03073-14)
Supplement: Supplemental material [file supp_53_4_1137__index.html]

Supplemental material 

# Mycobacterial DNA Extraction for Whole-Genome Sequencing from Early Positive Liquid (MGIT) Cultures

## Supplemental material

**Files in this Data Supplement:**

- Supplemental file 1 -

  Protocol S1 (Modified Nextera XT DNA sample preparation guide)

  PDF, 175K
