# Supplementary material for: Mycobacterial DNA Extraction for Whole-Genome Sequencing from Early Positive Liquid (MGIT) Cultures
Source: J Clin Microbiol. 2015 Mar 18;53(4):1137–43. doi: 10.1128/JCM.03073-14 (PMC4365189; doi:10.1128/JCM.03073-14)
Supplement: Supplemental material [file JCM.03073-14_zjm999094142so1.pdf]

Supplemental File 1: Modified Nextera XT DNA Sample Preparation Guide (Illumina, USA, Part # 15031942 rev. C, October 2012).

Required Kit content:

Box1

ATM Amplicon Tagment Mix

TD Tagment DNA Buffer

NPM Nextera PCR Master Mix

RSB Resuspension Buffer

HT1 Hybridization Buffer

Box 2

NT Neutralize Tagment Buffer (Room temperature)

DNA requirements:

5ul of genomic DNA 0.2ng/ul (1ng of DNA in total)

A. Tagmentation of DNA

1. Remove the ATM, TD, and DNA from -15° to -25°C storage and thaw on ice.
2. After thawing, ensure all reagents are adequately mixed by gently inverting the tubes 3–5 times, followed by a brief spin in a microcentrifuge.
3. Ensure that NT is at room temperature.
4. Add 10ul of TD Buffer to each well to be used in this assay.
5. Add 5ul of input DNA at 0.2ng/μl (1ng total) to each sample well of the plate.
6. Add 5ul of ATM to the wells containing input DNA and TD Buffer.
7. Using a multichannel pipette, gently pipette up and down 10 times to mix. Change tips between samples.
8. Cover the plate with Microseal 'B' and centrifuge briefly.
9. Place the plate in a thermocycler and run the following program:
   55°C 5 minutes
   10°C Hold
10. Once the sample reaches 10°C proceed immediately to Neutralization of Tagmentation reaction.

B. Neutralization of Tagmented DNA

1. Carefully remove the Microseal “B” seal and add 5ul of NT Buffer to each well of the plate with samples.
2. Using a multichannel pipette, gently pipette up and down 5 times to mix.
3. Briefly centrifuge the plate.
4. Incubate 5 minutes at room temperature.

C. Index PCR Setup

1. Select the correct idex 1 (i7) and index 2 (i5) for the number of libraries in the pooling set.
2. Remove NPM and the Index primers from -15° to -25°C storage and thaw on a bench at room temperature.
3. After all reagents are completely thawed, gently invert each tube 3–5 times to mix and briefly centrifuge the tubes in a microcentrifuge.
4. For 24 libraries arrange the index primers in the TruSeq Index Plate Fixture using the following arrangement:
   a. Arrange index 1 (i7) primers (orange caps) in order horizontally, so N701 is in column 1 and N706 is in column 6.
   b. Arrange index 2 (i5) primers (white caps) in order vertically, so S501 is in row A and S504 is in row D.
5. Add 15ul of NPM to each well of the plate with neutralized tagmented DNA. Change tips between samples.
6. Add 5ul of index 2 primers (white caps) to each column of the plate. To avoid index cross-contamination, discard the original white caps and apply new white caps provided in the kit.
7. Add 5ul of index 1 primers (orange caps) to each row of the plate. To avoid index cross-contamination, discard the original orange caps and apply new orange caps provided in the kit.
8. Seal the plate and centrifuge briefly.
9. PCR (**15 cycles**):

Index 2	5ul
Index 1	5ul

Nextera PCR MM (NPM)	15ul
DNA	25ul
Total Volume	50ul

10. PCR reaction conditions:

72°C	3 min	
95°C	30 s	
95°C	10 s	**<u>15 cycles</u>**
55°C	30 s	
72°C	30 s	
72°C	5 min	
10°C	Hold	

11. SAFE STOPPING POINT (keep plate at 2-8°C for up to 2 days).

D. Index PCR Clean-Up

1. Centrifuge the plate briefly to collect condensation.
2. Add 30ul (x0.6) of AMPure XP magnetic beads (Beckman Coulter, UK) to each well and vortex for 10 seconds (For 2x250 runs on the MiSeq, add 25ul (x0.5) of AMPure XP magnetic beads).
3. Incubate at room temperature for 5 minutes.
4. Transfer the plate to magnetic holder, incubate for 3 minutes and remove supernatant.
5. Wash the beads 2 times with 200µl of 80% ethanol. Incubate for 1 minute before removing the supernatant.
6. Air dry beads for 10-15 minutes at room temperature.
7. To elute add 26ul of RSB to each well of the plate and pipette up and down until beads are completely re-suspended. It is advisable to vortex the plate after beads are completely re-suspended for 30 seconds.
8. Incubate the plate for 5 minutes and briefly spin before putting plate on a magnet for 4 minutes.
9. Transfer 25ul of clean product to new plate.
10. SAFE STOPPING POINT (keep plate at -20°C).

E. Library QC

1. Measure library concentration using Qubit 2.0 Fluorometer (Life Technologies, US).
2. If bioanalyzer is available, check library size distribution by running 2ul of library on 2200 TapeStation Bioanalyzer (Agilent Technologies, USA). Record library average size (bp).

F. Library Normalization

1. Normalisation with and without bioanalyzer
    a. If bioanalyzer is available convert the library concentrations to nM using Qubit measurements (ng/ul) and Bioanalyzer measurements of the library's average size (bp) using the following formula:
    Library concentration, nM = (6(250/Library length, bp)*Library concentration, ng/ul*
    b. Calculate the volume of buffer required for each library to dilute it to 4nM or 10nM using the following formula:
    *Volume of buffer for dilution, ul = ((Library concentration, nM * volume of library for dilution, ul)/required library concentration, nM) - volume of library for dilution, ul*
    Note: Choose the highest possible normalization concentration based on concentration of your library.
    c. If bioanalyzer is not available use Qubit measurements (ng/ul) and normalize libraries to 1.6ng/µl, or 4ng/µl, to achieve 4nM or 10nM libraries respectively. (Conversion factor of 1ng/ul = 2.5nM) Note: If 1.6ng/µl cannot be achieved, proceed to magnetic beads normalization step of full Nextera XT protocol.
2. Normalize the concentration of each library using 10mM Tris-Cl buffer (pH 8.5) with 0.1% Tween. To prepare 10mM Tris-Cl with 0.1% Tween: (1) add 20ul of 1M Tris-Cl to 1980ul of H20 to get 10mM Tris-Cl; (2) add 10ul of 100% Tween to 990ul of 10mM Tris-Cl to get 1% Tween in 10mM Tris-Cl; (3) add 100ul of 1% Tween to 900ul of 10 mM Tris-Cl to get final solution of 10mM Tris-Cl with 0.1% Tween.
3. Vortex plate and spin briefly.
4. SAFE STOPPING POINT (keep plate at -20°C).

G. Library Pooling

1. If libraries were frozen, thaw the plate with normalized libraries and centrifuge briefly to collect the supernatant.
2. Heat the plate at 95°C for 5 minutes in PCR machine and shake the plate at 1800 rpm for 5 minutes.
3. Centrifuge the plate briefly to collect the supernatant.
4. Transfer 5ul of each normalized library to new 1.5ml Eppendorf tube.

H. Denaturing of Pooled Libraries

1. Prepare 1ml of fresh 0.2N NaOH (add 20ul of 10N NaOH to 980ul of H2O).
2. Combine 15ul of 4 or 10nM DNA pool with 15ul of 0.2N NaOH, vortex and centrifuge briefly.
3. Incubate for 5 minutes at room temperature to denature DNA into single strands.
4. To obtain 50pM of denatured pool:
   For 4nM pool: add 25ul of denatured DNA to 975ul of pre-chilled HT1.
   For 10nM pool: add 10ul of denatured DNA to 990ul of pre-chilled HT1.
5. To obtain 600ul of desired input concentration, dilute the 50pM DNA using the table below:

Final pool concentration	16pM	18pM	20pM
50pM denatured DNA	192ul	216ul	240ul
Pre-chilled HT1	408ul	384ul	360ul

6. Vortex the tube and spin briefly. Keep the pool on ice before combining with 12.5pM Phix control.

I. Prepare PhiX control

1. To dilute PhiX to 4nM, add 2ul of 10nM PhiX library to 3ul of 10mM Tris-Cl with 1% Tween 20.
2. Prepare 1ml of fresh 0.2N NaOH (add 20ul of 10N NaOH to 980ul of H2O) unless you already have some from denaturing of pooled libraries.
3. Combine 5ul of 4nM PhiX with 5ul of 0.2N NaOH, vortex and centrifuge briefly.

4. Incubate for 5 minutes at room temperature.
5. To obtain 20pM PhiX add 10ul of denatured PhiX to 990ul of pre-chilled HT1 (20pM PhiX could be stored at -20°C up to 3 weeks).
6. To obtain 12ul of 12.5pM PhiX, add 7.5ul of 20pM denatured PhiX to 4.5ul of HT1.

G. Combine DNA Pool and PhiX

1. To add 1% PhiX to the pool, combine 6ul of 12.5pM Phix to 594ul of denatured pool.
2. Vortex the tube and spin briefly.
3. Load pool into MiSeq cartridge.
